# Platelets Facilitate Wound Healing by Mitochondrial Transfer and Reducing Oxidative Stress in Endothelial Cells

**DOI:** 10.1155/2023/2345279

**Published:** 2023-02-20

**Authors:** Panshi Jin, Qiao Pan, Yize Lin, Yunqing Dong, Jie Zhu, Tao Liu, Weidong Zhu, Biao Cheng

**Affiliations:** ^1^General Hospital of Southern Theater Command of PLA, The First School of Clinical Medicine, Southern Medical University, Guangzhou 510010, China; ^2^General Hospital of Southern Theater Command of PLA, Guangzhou University of Chinese Medicine, Guangzhou 510010, China; ^3^Department of Plastic Surgery, The Second Affiliated Hospital of Kunming Medical University, Kunming 650000, China

## Abstract

As a critical member in wound healing, vascular endothelial cells (ECs) impaired under high levels of reactive oxygen species (ROS) would hamper neovascularization. Mitochondria transfer can reduce intracellular ROS damage under pathological condition. Meanwhile, platelets can release mitochondria and alleviate oxidative stress. However, the mechanism by which platelets promote cell survival and reduce oxidative stress damage has not been clarified. Here, first, we selected ultrasound as the best method for subsequent experiments by detecting the growth factors and mitochondria released from manipulation platelet concentrates (PCs), as well as the effect of manipulation PCs on the proliferation and migration of HUVECs. Then, we found that sonicate platelet concentrates (SPC) decreased the level of ROS in HUVECs treated with hydrogen peroxide in advance, increased mitochondrial membrane potential, and reduced apoptosis. By transmission electron microscope, we saw that two kinds of mitochondria, free or wrapped in vesicles, were released by activated platelets. In addition, we explored that platelet-derived mitochondria were transferred to HUVECs partly by means of dynamin-dependent clathrin-mediated endocytosis. Consistently, we determined that platelet-derived mitochondria reduced apoptosis of HUVECs caused by oxidative stress. What is more, we screened survivin as the target of platelet-derived mitochondria via high-throughput sequencing. Finally, we demonstrated that platelet-derived mitochondria promoted wound healing in vivo. Overall, these findings revealed that platelets are important donors of mitochondria, and platelet-derived mitochondria can promote wound healing by reducing apoptosis caused by oxidative stress in vascular endothelial cells. And survivin is a potential target. These results further expand the knowledge of the platelet function and provide new insights into the role of platelet-derived mitochondria in wound healing.

## 1. Introduction

Wound healing is a complex and orderly process, which is divided into several consecutive processes including hemostasis, inflammation, proliferation, and maturation remodeling [1]. As a process of the proliferation phase, angiogenesis is critical for efficient wound healing by delivery of adequate nutrients and oxygen [2]. Microvascular endothelial cells (ECs) are arranged in the inner wall of blood vessels and are the initial cells involved in forming new blood vessels during wound healing [3]. The normal process of wound healing depends on the integrity and function of vascular endothelial cells [4]. However, under some physiological or pathological conditions, endothelial cells can be dysfunctional, and apoptosis appears due to the oxidative stress triggered by excessive reactive oxygen species (ROS), while low-level ROS is present and necessary for vascularization [4]. The control of the oxidative stress state of endothelial cells may better regulate the angiogenesis in the process of wound healing, avoid vascular damage or excessive proliferation, and thus accelerate wound healing.

Mitochondria play a critical role in the regulation of metabolic processes that govern the function and fate decision of stem cells of an adult, embryonic/pluripotent, or cancer origin [5–7]. Recent evidence suggests that mitochondria play a critical role in controlling wound healing. Evidence shows a requirement of mitochondrial respiration in vascular endothelial cells for neoangiogenesis during development, tissue repair, and cancer [8]. Surprisingly, there is growing evidence in the control of mitochondria cross cells [9–11]. It has been reported that intercellular mitochondrial transfer can not only occur in the central nervous system, cardiovascular system, and respiratory system but also to contribute to multifunctional cellular activity and thereby have an impact on tumor therapy resistance and inflammation regulation [10]. The transferred mitochondria can reduce intracellular ROS damage under pathological condition [12–14]. However, we do not know whether mitochondrial transfer can similarly reduce oxidative stress in wound healing, especially in endothelial cells, thus promoting wound healing.

Interestingly, the mitochondria released by platelets are also gradually recognized. Platelets are special cells or cell fragments fall off from megakaryocytes stationed in the bone marrow [15]. Platelets can release mitochondria into membrane-wrapped particles with a size of more than 500 nanometers or as free organelles [16]. However, the molecular mechanism and consequences of this phenomenon are unclear. Two studies have described that mitochondria released by activated platelets can be absorbed by neutrophils or islets, triggering “immune” or “healing” responses, respectively [16, 17]. The latest study found that these mitochondria released by platelets can be transferred to mesenchymal stem cells and promote the vascular induction ability of mesenchymal stem cells through metabolic reprogramming [18]. However, there is no report of platelet mitochondria promoting wound healing directly. Several recent studies have showed that PCs could alleviate oxidative stress, including in skeletal muscle contusion, gingival fibroblasts, and human spermatozoa, and ameliorate nephrotoxicity [19]. At the same time, platelets and their mitochondria may also be the sources of ROS [20–22]. Considering the role of mitochondrial transfer in reducing cell injury, it is worth considering whether the mitochondria released by platelets play a role in the process of platelet concentrates reducing oxidative stress.

Here, we explored whether platelet mitochondria can be transferred to ECs to reduce the apoptosis induced by oxidative stress and thereby to promote wound healing. Besides, we investigated the mechanism of platelet mitochondria transferred to endothelial cells and then reduced EC apoptosis. What is more, our study described the characteristics of several manipulation PCs and provided a new promising donor for mitochondrial transplantation. Overall, our findings provide a new explanation for platelet concentrates in the treatment of wound healing and new practice to promote wound healing by regulating oxidative stress in endothelial cells.

## 2. Materials and Methods

### 2.1. Preparation of Platelet Concentrates

The study got approval from the Research Ethics Commission of General Hospital of Southern Theater Command of PLA. Platelet concentrates were collected from the venous blood of health volunteers. EDTA was used to prevent blood clotting. After the whole blood was collected, it would be centrifuged for 300 x g (10 min) at 22°C to obtain the buffy coat layer and plasma. Another centrifuge for 900 x g (15 min) at 22°C made platelets precipitated. According to the platelet count in the whole blood, the pellet was resuspended in the plasma or specific buffer. The platelets were counted by a hematology analyzer (Mindray, Shen Zhen, China).

### 2.2. Manipulation of Platelet Concentrates

There were four methods to activate platelet concentrates. Briefly, frozen-thawed platelet concentrates (FTPC) were obtained by three freeze (−80°C) and thaw (37°C) cycles to lyse cell membranes [23]. The FTPC were centrifuged at 4000 x g for 20 min at 4°C to remove cell debris. The supernatant was used immediately or stored frozen at -80°C. Thrombin platelet concentrates (TPC) were obtained by adding 142.8 U/mL thrombin (final concentration) (T832140-1KU, Macklin, China) to induce platelet activation [24]. Then, the mixture was incubated in water at 37°C for 30 min. The fibrin clot and cell debris were removed by centrifugation 4000 x g for 20 min at 4°C. Sonicate platelet concentrates (SPC) were obtained by sonicating PC in a centrifuge tube for 35 cycles (5 seconds on and 5 seconds off for each cycle) [25]. The SPC were centrifuged at 4000 x g for 20 min at 4°C to remove cell debris. Light platelet concentrates (LPC) were obtained by being exposed to 808 nm laser from a distance of 10 cm for 30 min [26]. Consistent with the above description, cell debris was removed by centrifugation (4000 x g for 20 min at 4°C). Sterile heparin (40 U/mL, final concentration) (H811552-100K, Macklin, China) was added to all PCs prior to culturing cells to prevent the generation of gel. All supernatant samples were aliquoted and stored at −80°C until use.

### 2.3. Growth Factor Detection

Following the manufacturer's protocol, concentrations of platelet-derived growth factor ((PDGF)-BB, bsk11090), epidermal growth factor (EGF, bsk11025), fibroblast growth factor (FGF-b, bsk11032), and transforming growth factor (TGF-*β*1, bsk11021) were quantified by enzyme-linked immunosorbent assay (Bioss, China).

### 2.4. Isolation of PC Mitochondria and Inhibition of Mitochondrial Respiration

Mitochondria were separated from the above-mentioned PC supernatant as previously described [27]. Briefly, the supernatant was centrifuged at 10000 x g for 10 min at 4°C to pellet the mitochondrial extract. The mitochondrial extract was then resuspended in mitochondrial isolation buffer (MIB, 70 mM sucrose, 210 mM mannitol, 5 mM HEPES, 1 mM EGTA, and 0.5% (*w*/*v*) fatty acid-free BSA, pH 7.2). Then, it was layered on a 15% Percoll layer (15% Percoll, 10% sucrose 2.5 M, 75% MIB). The tube was then centrifuged at 21000 x g at 4°C for 10 min. The pellet was washed with MIB at 13000 x g at 4°C for 10 min.

To inhibit mitochondrial respiration, we selected the inhibitor of ATP synthase oligomycin (ab141829-1 mg, Abcam, Britain) and uncoupler FCCP (C2920-10MG, Sigma, USA). According to the description of previous research [18], platelets were treated for 1 h with 2 *μ*M oligomycin and 2 *μ*M FCCP and then washed 3 times (900 x g at 22°C for 15 min) in DMEM containing heparin (40 U/mL) to avoid platelet activation and aggregation.

### 2.5. Seahorse Analysis

The oxygen consumption rate (OCR) of isolated mitochondria, indicative of mitochondrial respiration, was assessed using the Seahorse XF96 Analyzer (Agilent, USA) [18, 27]. Briefly, isolated mitochondria were resuspended in MIB. The OCR was evaluated using the Agilent Seahorse XF Cell Mito Stress Test, with sequential additions of ADP (4 mM, final) (A2754-1G, Sigma, USA), oligomycin (2.5 *μ*g/mL, final), FCCP (4 *μ*M, final), and rotenone (4 *μ*M, final) (R8875-1G, Sigma, USA). Results were analyzed using Seahorse Wave Desktop Software (Agilent, USA).

### 2.6. Western Blot Assay

Cells and mitochondria were washed with cold PBS and lysed with RIPA buffer (V900854-100ML, Sigma, USA) containing protease inhibitor cocktails (P1045, Beyotime, China) for 30 min at 4°C. Lysates were centrifuged at 14000 x g for 15 min at 4°C. Lysates were diluted at a ratio of 1 : 4 with protein loading buffer (5 x) (P0015L, Beyotime, China) and denatured at 95°C for 10 min. The protein extracts were separated by SDS-PAGE and transferred onto PVDF membranes (Millipore, USA). The membrane was blocked with Protein Free Rapid Blocking Buffer (PS108P, Epizyme, China) for 30 min at 22°C and incubated overnight at 4°C with primary antibodies. The primary antibodies for the blots were as follows: Tom 20 (1 : 1000, 42406S, CST), complex IV (COX-IV, 1 : 1000, 4844S, CST), TGF-*β* (1 : 1000, 3711S, CST), and cleaved caspase 3 (1 : 1000, 9664S, CST). Then, the membranes were incubated with secondary antibodies coupled with horseradish peroxidase (1 : 3000, 7074, CST) for 1 h at room temperature. As internal controls, antibody GAPDH (1 : 1000, 5174, CST) was used. For isolated mitochondria, we normalize the bands by controlling the consistent number of platelets in each sample. The chemiluminescence was detected with enhanced chemiluminescence reagent (ECL, WBKLS0500, Merck Millipore, USA) from three times experiments.

### 2.7. Cell Cultures

Human umbilical vein endothelial cells (HUVECs) were obtained from Shanghai Institute of Cell Biology, Chinese Academy of Sciences, supplemented with 10% (*v*/*v*) fetal bovine serum (FBS) and 1% antibiotics. HUVECs at passages 3 to 6 were used for all experiments. HUVECs were incubated in a humidified atmosphere of 5% CO_2_ at 37°C.

### 2.8. Migration and Viability Assays

Scratch wound assays were used to evaluate cell migration. Briefly, 2 × 10^5^ cells were plated into 24 well plate (Corning, USA). HUVEC monolayers were scratched with a 200 *μ*L tip for three times in every well. After washed, 5% manipulation PC supernatant was added into the well. Wound closure was analyzed at 24 h after scratching.

The HUVECs were seeded in 96 well at a density of 1 × 10^4^ cells/well and allowed to attach overnight. The next day, the medium was replaced with supplemented medium with or without drug treatments. After 24 h, the cell proliferation was assayed using Cell Counting Kit-8 (CCK-8, Dojindo, Japanese) following the manufacturer's instructions.

### 2.9. Cell Apoptosis and ROS Measurement by Flow Cytometry

Cell apoptosis and ROS levels were evaluated using an Annexin V-FITC/PI Apoptosis Detection Kit (V13241, Invitrogen, USA) or DCFH-DA probes (S0033S, Beyotime, China) separately according to the manufacturer's instruction. Briefly, the HUVECs were seeded in 6 well at a density of 2 × 10^5^ cells/well and allowed to attach overnight. The next day, the medium was replaced with serum-free medium added 1.2 mM H_2_O_2_ for 24 h or not. Then, the cells were treated with drug. For cell apoptosis detecting, cells were coincubated with Annexin V-FITC and then propidium iodide for 15 min at 22°C and then detected by flow cytometry. For ROS detecting, cells were coincubated with DCFH-DA probes (10 *μ*mol/L) at 37°C for 30 min and collected by centrifugation. After resuspended with PBS, DCFH-DA-labeled cells were further detected by flow cytometry.

### 2.10. Mitochondrial Membrane Potential Assay

Mitochondrial membrane potential was evaluated using enhanced mitochondrial membrane potential assay kit with JC-1 (C2003S, Beyotime, China). Briefly, the HUVECs were seeded in 96 well at a density of 1 × 10^4^ cells/well and allowed to attach overnight. The next day, the medium was replaced with serum-free medium added 1.2 mM H_2_O_2_ for 24 h or not. Then, the cells were treated with drug. For detecting, cells were coincubated with JC-1 probes as recommended concentration at 37°C for 20 min and washed by DMEM. Cells were observed and photographed through fluorescence microscope.

### 2.11. Transmission Electron Microscopy

Sonicate platelet concentrates (SPC) were obtained by sonicating PCs in a centrifuge tube for 35 cycles (5 seconds on and 5 seconds off for each cycle). The SPC were centrifuged at 10000 x g for 20 min at 4°C to precipitate mitochondrial and cell debris. The sediment was washed in PBS prior to be fixed with 2.5% glutaraldehyde for 24 h at 4°C. Then, the samples were gradually dehydrated in graded series of acetone (70%, 80%, 90%, and 100% for 15 min each) at room temperature. Resin was used to infiltrate and embed samples at room temperature. After cut, the mitochondrial samples were visualized using transmission electron microscope.

### 2.12. RNA Sequencing

Total RNA was extracted from HUVECs by Trizol reagent. Then, we constructed cDNA library and aligned it with the human reference gene. DESeq2 was performed for differential gene expression. After revising *p* value, we chose *p* value ≤ 0.05 and |log2foldchange| > 1 as standard for significant difference. Differential genes expression was then subjected to GO analysis, functional annotation analysis, and KEGG database analysis to determine the functional pathways.

### 2.13. Survivin Si-RNA Transfection

Si-RNA and NC mimics were purchased from Guangzhou IGE Biotechnology. The HUVECs were seeded in 6 well at a density of 1 × 10^6^ cells/well and allowed to attach overnight. To transfect HUVECs, Si-RNA or NC was diluted in Opti-MEM (31985070, Invitrogen, USA) and then blended with the mixture of Lipofectamine® RNAiMAX (13778-150, Invitrogen, USA) and Opti-MEM. After incubated for 20 min at room temperature, the mixture was added to each well.

### 2.14. Mouse Cutaneous Wounds

All experiments were performed according to the institutional guidelines for animal care and were approved by the Research Ethics Commission of General Hospital of Southern Theater Command of PLA.

Full thickness cutaneous wounds were created on the back of 8-week-old male C57BL/BL6 mice, aged from 6 to 8 weeks. Briefly, animals were anesthetized by 1% pentobarbital sodium solution (0.006 mL/g). The dorsal surface was shaved with an electric clipper and sterilized with 70% alcohol. Full-thickness 10 mm diameter cutaneous wounds were created on the back of each mouse (2 wounds on each side of the midline). Immediately after the skin injuries, each wound was injected with 100 *μ*L of SPC or SPC + Int (inhibitor of mitochondrial, oligomycin, and FCCP). Control wounds were injected with PBS. The wounds during the process of concrescence were recorded on days 0, 3, 7, and 10.

### 2.15. Wound Closure Analysis

Photographs were taken on days 0, 3, 5, and 10 after surgery. Photographs were taken with a ruler that was used to normalize the wound sizes. Wound closure was quantified using Image J software to evaluate of the wound reepithelialization on days 3, 5, 7, and 10 postsurgery and was calculated as follows: [(1 − wound area on day *X*)/wound area on day 0] × 100%. Wound samples were taken on days 3, 5, 7, and 10. The wounds were excised, dissected, and immediately fixed in 4% paraformaldehyde for next analyses. The samples were embedded in paraffin, sectioned at 5 *μ*m, and stained with hematoxylin and eosin (H&E) and Masson staining for microscopic observation.

### 2.16. Immunohistochemical Evaluation of Angiogenesis in Mouse Wounds

Immunohistochemical measurements of angiogenesis were performed on days 7 and 10 postsurgery. Mouse wounds were used to make paraffin sections as above. Tissue sections were deparaffinized, rehydrated, and pretreated for heat-mediated antigen retrieval. Sections were then incubated with anti-mouse CD31 antibody (1 : 1000, ab124432, Abcam, Britain) followed by incubated with biotinylated secondary antibodies (ZSGB-Bio, China) at room temperature for 30 min. All sections were colored by diaminobenzidine solution (ZSGB-Bio, China), and the nuclei were counterstained with hematoxylin (Beyotime, China). Then, the sections were photographed using a microscope.

### 2.17. Statistical Analysis

Data were presented as means ± SD. One-way ANOVA was used to compare three groups or more when the data satisfied the normal distribution and chi-square. All data were analyzed by using GraphPad Prism 7.0 (GraphPad Software, Inc.). *p* < 0.05 was considered statistically significant.

## 3. Results

### 3.1. Manipulation of Platelet Concentrates and Mitochondria Release

To characterize whole blood and platelet concentrates, we counted platelets by a hematology analyzer. Platelets were counted in the whole blood for 240 × 10^9^ cells/L and in the PCs for 1008 × 10^9^ cells/L. Consistent with common recommendation, the number of platelets in PCs is 3-5 times higher than that in whole blood.

**Figure 1:**
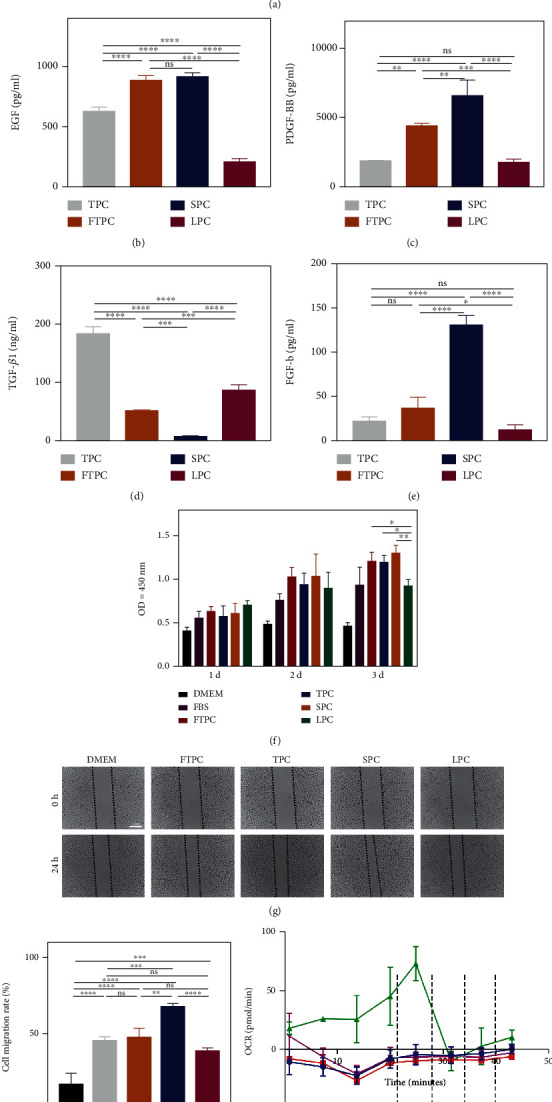
Comparison of manipulation platelet concentrates prepared by different methods. (a) The procedure of preparing platelet concentrates and manipulation platelet concentrates. (b–e) Enzyme-linked immunosorbent assay was used to detect the content of EGF, PDGF-BB, TGF-*β*1, and FGF-b in manipulation platelet concentrates. (f) CCK-8 assay was used to determine the cell viability of HUVECs treated with manipulation PCs. (g) Representative images of the wound closure of HUVECs treated with manipulation PCs at 0 h and 24 h and (h) statistical analysis of migration area (%) in scratch assay. (i) Oxygen consumption rate of mitochondria extracted from manipulation PCs (Adding ADP, oligomycin, FCCP, and rotenone in chronological order) and (j) Western blot showed the protein levels of COX IV and Tom 20. The data in the figures represent the mean ± SD. Significant differences between groups are indicated as ^∗^*p* < 0.05, ^∗∗^*p* < 0.01, ^∗∗∗^*p* < 0.001, ^∗∗∗∗^*p* < 0.0001.

In order to explore a more suitable manipulation method, we chose thrombin, ultrasound, repeated freeze/thaw, and light to activate platelet concentrates (Figure 1(a)). The release of growth factors is an important mean for platelets to promote tissue regeneration. In order to characteristic of several manipulation platelet concentrates, we detected the contents of PDGF-BB, FGF-b, EGF, and TGF-*β*1 in the supernatant (Figures 1(b)–1(e)). Significantly higher levels of PDGF-BB, FGF-b, and EGF and a lower concentration of TGF-*β*1 were measured in SPC compared to other manipulation PCs (Figures 1(b)–1(e)). FTPC contained similar growth factors with SPC, except for b-FGF (Figures 1(b)–1(e)). For TPC, it released moderate concentrations of growth factors generally and higher concentrations of TGF-*β*1 especially (Figures 1(b)–1(e)). LPC seem to be a weak manipulation method, releasing lower concentrations of EGF, PDGF-BB, and FGF-b (Figures 1(b), 1(c), and 1(e)). In addition, we explored the existence forms of TGF-*β*1 in several manipulation platelet concentrates. The results showed that the TGF-*β*1 in several manipulation platelet concentrates existed in mature form (Figures S1). We next investigated whether these manipulation PCs exerted different effects on cells in vitro. Unlike the difference of growth factors, we found that several PCs had similar effects on cell proliferation, except for LPC (Figure 1(f)). However, SPC were more obvious in promoting cell migration than others (Figures 1(g) and 1(h)).

Platelets can release mitochondria into membrane-wrapped particles with a size of more than 500 nanometers or as free organelles. In order to explore the effects of several manipulation methods on the release of mitochondria from platelets, we precipitated the mitochondria by centrifuge and measured mitochondria yield and oxygen consumption rate. First, we used Western blot to determine which method would make most mitochondria yield. Tom 20 is the most important receptor subunit in TOM complex, which is located in the outer membrane of mitochondria. COX IV is a subunit of COX which locates in the inner membrane of mitochondria and is an enzyme complex of mitochondrial respiratory chain. The expression of Tom 20 and COX IV showed that SPC maximized the release of mitochondria from platelets (Figure 1(j)). Given that oxygen consumption is a major link to adenosine triphosphate (ATP) production in mitochondria, we investigated the bioenergetics of mitochondria precipitated from several manipulation PCs via detecting OCR. Consistent with the result of Western blot, we saw the mitochondria derived from the SPC exhibiting higher basal OCR than other samples (Figure 1(i)). The results suggested that ultrasound is a more appropriate method to activate platelets to release respiratory mitochondria.

### 3.2. SPC Protect HUVECs from Oxidative Damage

**Figure 2:**
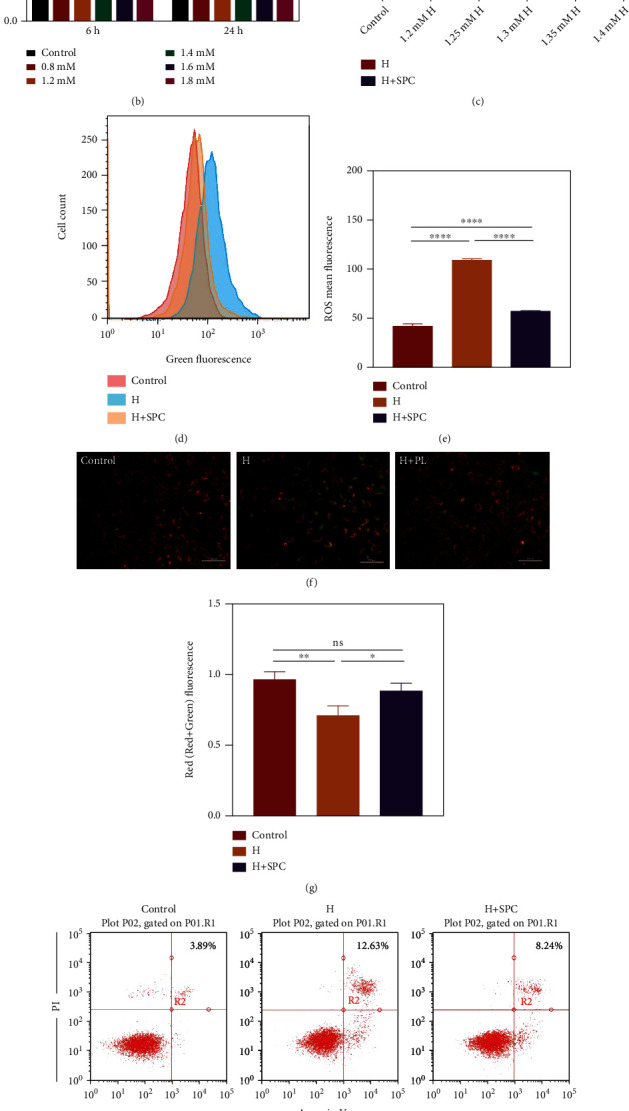
The effect of SPC on apoptosis in HUVECs. (a) Representative images of HUVECs treated with H_2_O_2_ for 6 h. (b) CCK-8 assay was used to determine the cell viability of HUVECs treated with H_2_O_2_ for 6 h and 24 h. (c) CCK-8 assay was used to determine the cell viability of HUVECs incubated with SPC after treated with H_2_O_2_ for 24 h. (d) Flow cytometric analysis of the ROS level in HUVECs incubated with SPC after treated with H_2_O_2_ for 24 h and (e) statistical analysis of ROS mean fluorescence. (f) Representative images of JC-1 staining of HUVECs incubated with SPC after treated with H_2_O_2_ for 24 h and (g) statistical analysis of Red/(Red+Green) fluorescence. (h) Flow cytometric analysis of apoptosis in HUVECs incubated with SPC after treated with H_2_O_2_ for 24 h and (i) the percentages of apoptotic cells (upper-right) based on total cell population. The data in the figures represent the mean ± SD. Significant differences between groups are indicated as ^∗^*p* < 0.05, ^∗∗^*p* < 0.01, ^∗∗∗^*p* < 0.001, ^∗∗∗∗^*p* < 0.0001.

In order to detect the effect of SPC on HUVECs treated with H_2_O_2_, the H_2_O_2_-induced oxidative injury model of HUVECs was established. First, we performed experiments to determine the concentration and time of H_2_O_2_ for treating cells. The cell viability and the cell morphology were the main index to estimate H_2_O_2_-induced HUVECs injury. With the increase of the concentration of H_2_O_2_ and the prolongation of treatment time, the cell proliferation activity decreased and the cell morphology changed (Figures 2(a) and 2(b)). We selected 24 h and 1.2 mM as the appropriate time and concentration of H_2_O_2_ to avoid a large number of cells death or insufficient damage. To determine the protective role of SPC on HUVECs of oxidative damage, we examined cell viability by CCK8 (Figure 2(c)). Within certain concentration range of H_2_O_2_, cell proliferation ability was increased by SPC (Figure 2(c)). Without H_2_O_2_ or too high concentration of H_2_O_2_ would lead to different results that there was no significant difference of cell proliferation ability between two groups (Figure 2(c)). To further confirm the protective role, we examined ROS levels by flow cytometry. SPC treatment significantly decreased ROS levels in H_2_O_2_-treated cells (Figures 2(d) and 2(e)). Oxidative stress is also often associated with mitochondrial impairment. We used JC-1 to measure mitochondrial membrane potential (MMP) that is an indicator of mitochondrial state. Consistent with the above results, SPC significantly increased mitochondrial membrane potential in H_2_O_2_-treated cells (Figures 2(f) and 2(g)). Apoptosis is the main death mode of endothelial cells under oxidative stress. Therefore, we further tested whether SPC alleviate apoptosis of endothelial cells. Flow cytometry assay showed that SPC significantly alleviated the apoptotic of HUVECs (Figures 2(h) and 2(i)).

### 3.3. Platelet-Derived Mitochondria in SPC Are Transferred to HUVECs

**Figure 3:**
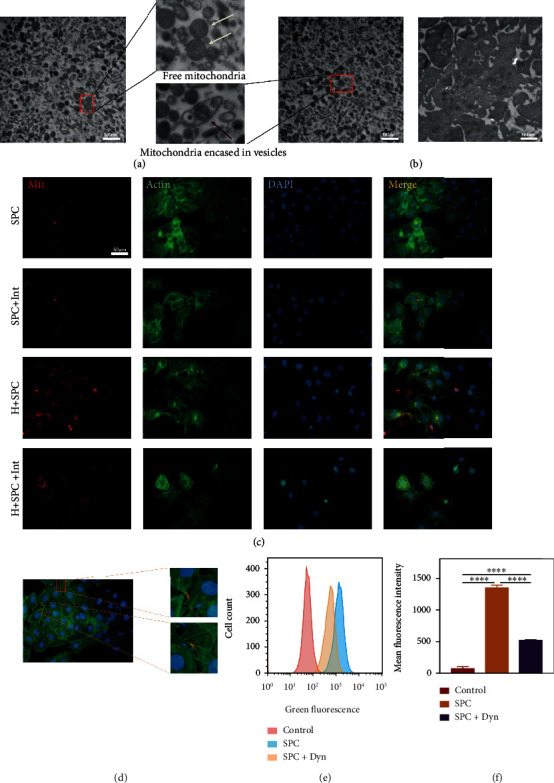
Platelet-derived mitochondria in SPC were taken up by HUVECs. (a) TEM was used to observe the mitochondria released by platelets treated with ultrasound. The white arrow pointed the exposed mitochondria, and the red arrow pointed to the membrane-wrapped mitochondria. (b) TEM was used to observe mitochondria within nonactivated platelets. (c, d) Representative images of HUVECs treated with H_2_O_2_ for 24 h after incubation with SPC or SPC following exposure to mitochondrial inhibitor and previously labeled with Mito Tracker Red. “Mit” labeled the mitochondria. “Actin” labeled the cytoskeleton. “DAPI” labeled the cell nucleus. (e) Flow cytometric analysis of the transfer of platelet-derived mitochondria in SPC to HUVECs previously treated with dynasore, an inhibitor of dynamin-dependent clathrin-mediated endocytosis, and (f) statistical analysis of mean fluorescence intensity. The data in the figures represent the mean ± SD. Significant differences between groups are indicated as ^∗^*p* < 0.05, ^∗∗^*p* < 0.01, ^∗∗∗^*p* < 0.001, ^∗∗∗∗^*p* < 0.0001.

We observed the released mitochondria from platelets by transmission electron microscopy (TEM) (Figure 3(a)). Consistent with previous reports, TEM analysis revealed that there were two kinds of “mitochondria-like” double-membrane structures: free or encapsulated (Figure 3(a)). Meanwhile, we found that nonactivated platelets contain 1-3 mitochondria (Figure 3(b)). To determine whether mitochondria transfer from SPC to HUVECs, we cocultured HUVECs for 24 h with labeled SPC with MitoTracker® Deep Red FM (Figure 3(c)). We next evaluated the presence of platelet-derived mitochondria in HUVECs using fluorescence microscopy (Figure 3(c)). Then, we investigated whether mitochondria transfer from SPC to HUVECs was disturbed by impairing function of platelet-derived mitochondria. We cocultured HUVECs for 24 h with labeled SPC with MitoTracker® Deep Red FM and previously treated with oligomycin and FCCP (SPC + Int) to inhibit mitochondrial respiration. Microscopy analysis showed the number of platelet-derived mitochondria taken up by HUVECs was similar for either SPC or SPC + Int (Figure 3(c)). In addition, HUVECs treated with hydrogen peroxide absorbed more platelet-derived mitochondria. Interestingly, we found a strange phenomenon that platelet-derived mitochondria distributed in the synapse of HUVECs (Figure 3(d)). This seems to mean that the mitochondria experienced a second transfer in the HUVECs. We then wondered the mechanism by which HUVECs internalized platelet-derived mitochondria and found that dynasore, an inhibitor of endocytosis, suppressed this transfer (Figures 3(e) and 3(f)).

### 3.4. Platelet-Derived Mitochondria in SPC Protect HUVECs from Oxidative Damage

**Figure 4:**
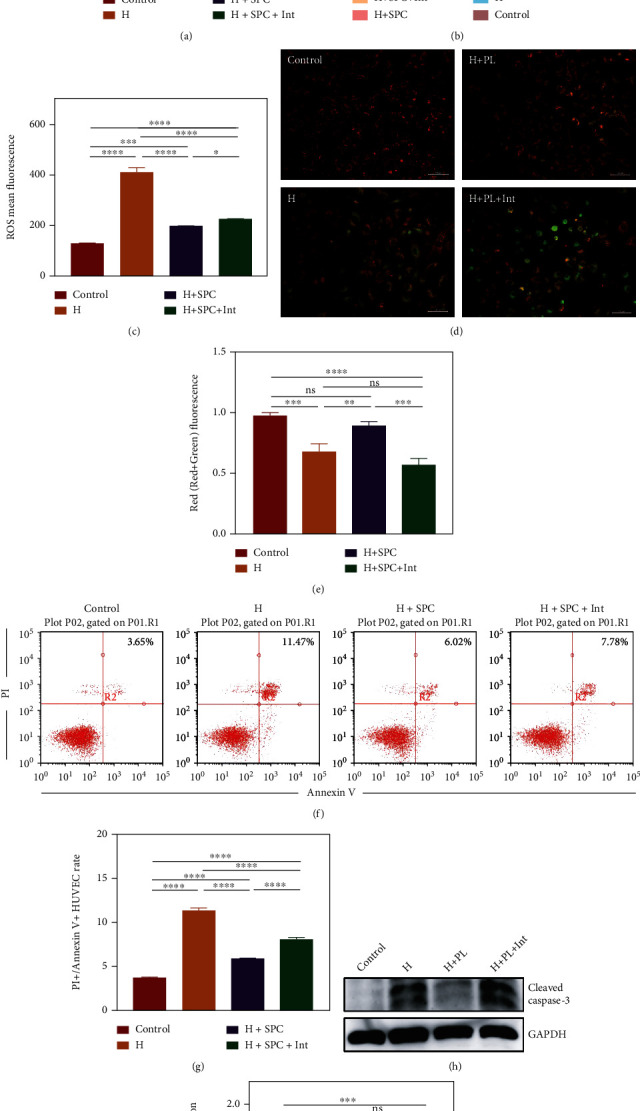
The effect of platelet-derived mitochondria in SPC on apoptosis in HUVECs. (a) CCK-8 assay was used to determine the cell viability of HUVECs incubated with SPC or SPC + Int after treated with H_2_O_2_ for 24 h. (b) Flow cytometric analysis of the ROS levels in HUVECs incubated with SPC or SPC + Int after treated with H_2_O_2_ for 24 h and (c) statistical analysis of ROS mean fluorescence. (d) Representative images of JC-1 staining of HUVECs incubated with SPC or SPC + Int after treated with H_2_O_2_ for 24 h and (e) statistical analysis of Red/(Red+Green) fluorescence. (f) Flow cytometric analysis of apoptosis in HUVECs incubated with SPC or SPC + Int after treated with H_2_O_2_ for 24 h and (g) the percentages of apoptotic cells (upper-right) based on total cell population. (h) Western blot showed the protein level of cleaved caspase 3 in HUVECs and (i) statistical analysis of the quantification of cleaved caspase 3 expression. The data in the figures represent the mean ± SD. Significant differences between groups are indicated as ^∗^*p* < 0.05, ^∗∗^*p* < 0.01, ^∗∗∗^*p* < 0.001, ^∗∗∗∗^*p* < 0.0001.

In order to explore the function of mitochondria, we used inhibitors, oligomycin, and FCCP, to inhibit the ATP synthase of platelet-derived mitochondria. Then, we cocultured H_2_O_2_-treated HUVECs with SPC or SPC + Int for 24 h. We examined cell viability by CCK8 to initially determine the rescue function of platelet-derived mitochondria (Figure 4(a)). The result showed that SPC increased cell viability, and platelet-derived mitochondria inhibited in SPC + Int reduced this effect (Figure 4(a)). To further confirm the protective role of platelet-derived mitochondria, we examined ROS levels in HUVECs by flow cytometry (Figures 4(b) and 4(c)). The results showed that SPC decreased ROS levels in HUVECs, and platelet-derived mitochondria inhibited in SPC + Int reduced this effect (Figures 4(b) and 4(c)). We also measured mitochondrial membrane potential to evaluate the effect of exogenous mitochondria on HUVEC mitochondria impaired by oxidative damage (Figures 4(d) and 4(e)). Consistent with previous results, the decrease of mitochondrial membrane potential was prevented by SPC (Figures 4(d) and 4(e)). Surprisingly, platelet-derived mitochondria inhibited in SPC + Int made the function of mitochondrial of H_2_O_2_-treated HUVECs worse (Figures 4(d) and 4(e)). Further, we tested whether platelet-derived mitochondria alleviated apoptosis of endothelial cells (Figures 4(f) and 4(g)). Flow cytometry assay showed that SPC significantly alleviated HUVEC apoptosis, and platelet-derived mitochondria inhibited in SPC + Int reduced this effect (Figures 4(f) and 4(g)). Caspase-3 has been identified as a key mediator of apoptosis in cells which suffered oxidative damage. Similarly, the caspase-3 activity was significantly increased in the SPC + Int (Figures 4(h) and 4(i)). These results suggested that the effects of SPC decreasing apoptosis in endothelial cells were partly mediated by platelet-derived mitochondria transferred to HUVECs.

### 3.5. Platelet-Derived Mitochondria in SPC Increase HUVEC Survivin Expression

**Figure 5:**
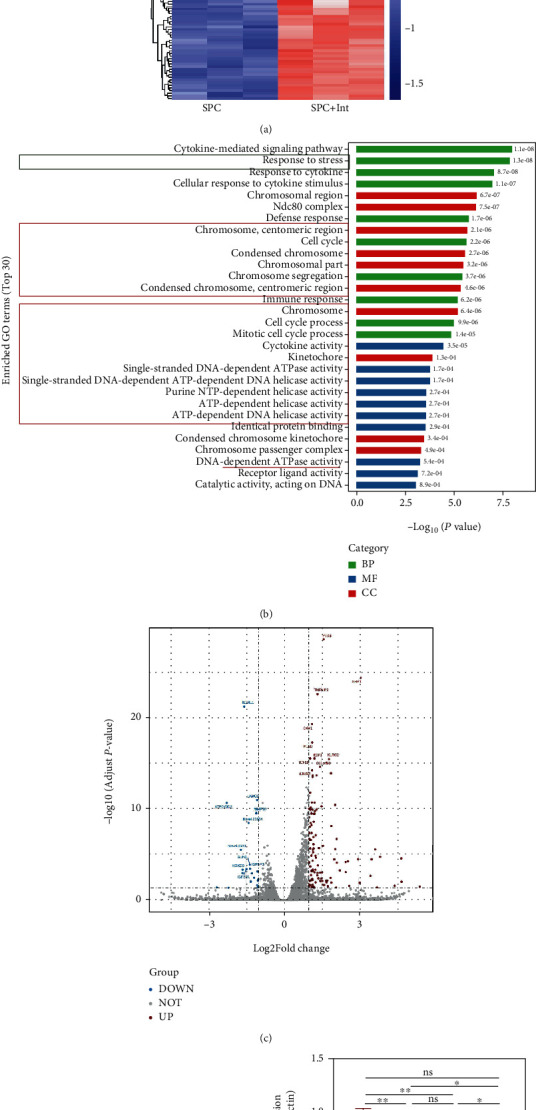
Platelet-derived mitochondria in SPC increased HUVEC antiapoptosis gene survivin expression. (a) The heatmap of RNA-seq in the HUVECs treated with H_2_O_2_ for 24 h and then 24 h incubation with SPC or SPC + Int. (b) The GO enrichment analysis of the differential genes of HUVECs in two groups. (c) The volcano plot showed differential genes of HUVECs in two groups. (d) Western blot showed the protein level of survivin in HUVECs treated with H_2_O_2_ for 24 h and then 24 h incubation with SPC or SPC + Int and (e) statistical analysis of the quantification of survivin expression. The data in the figures represent the mean ± SD. Significant differences between groups are indicated as ^∗^*p* < 0.05, ^∗∗^*p* < 0.01, ^∗∗∗^*p* < 0.001, ^∗∗∗∗^*p* < 0.0001.

In order to clarify the effect of platelet-derived mitochondria transplantation on HUVEC transcriptomic profile, we collected HUVECs treated with H_2_O_2_ and SPC or SPC + Int and performed high-throughput RNA sequencing (RNA-seq). We observed that differences in most identified mRNAs are significant, with 117 upregulated and 22 downregulated genes in SPC compared with SPC + Int (Figures 5(a) and 5(b)). Surprisingly, GO Enrichment results revealed a significant upregulation in pathways associated with stress and cellular proliferation, including CDC, CDK, E2F, and G2M target signaling (Figure 5(b)). After further screening differential genes, we found that the antiapoptosis gene, survivin, was upregulated in SPC. Combined with our analysis of the overall differential genes, platelet-derived mitochondria may improve the proliferation of endothelial cells and resist apoptosis. We further verified the expression change of survivin in HUVECs influenced by mitochondrial using Western blot (Figures 5(d) and 5(e)). Consistent with high-throughput sequencing results, platelet-derived mitochondria increased the expression of survivin (Figures 5(d) and 5(e)).

### 3.6. Survivin Mediates the Protective Effect of Platelet-Derived Mitochondria in SPC on HUVECs

**Figure 6:**
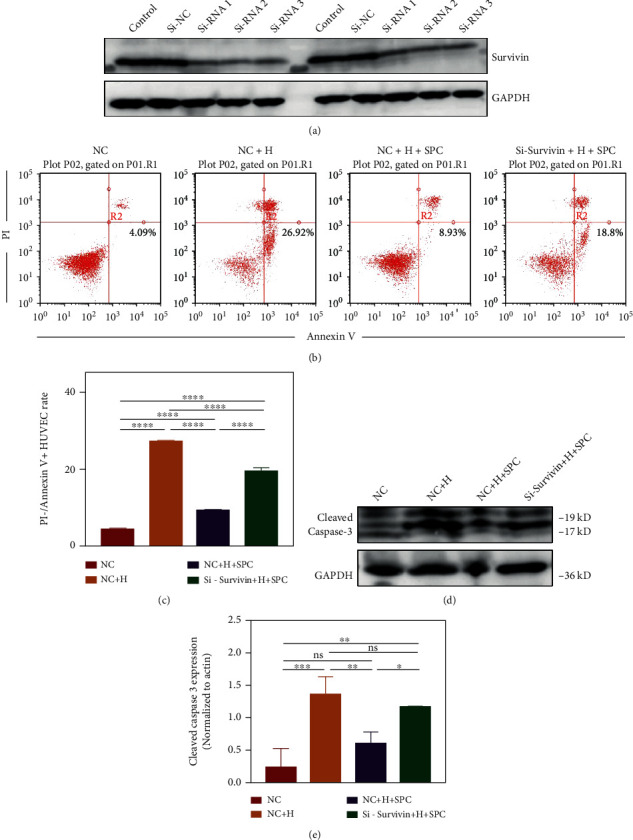
Survivin mediated the protective effect of platelet-mitochondria in SPC on HUVECs. (a) Western blot showed the protein level of survivin in HUVECs treated with si-RNA or si-NC. (b) Flow cytometric analysis of apoptosis in HUVECs and (c) the percentages of apoptotic cells (bottom-right) based on total cell population. (d) Western blot showed the protein level of cleaved caspase 3 in HUVECs and (e) statistical analysis of the quantification of cleaved caspase 3 expression. The data in the figures represent the mean ± SD. Significant differences between groups are indicated as ^∗^*p* < 0.05, ^∗∗^*p* < 0.01, ^∗∗∗^*p* < 0.001, ^∗∗∗∗^*p* < 0.0001.

To explore the function of survivin, three small interfering RNAs (siRNAs) were designed to decrease the expression of survivin. Western blot results indicated that three siRNAs equally inhibited survivin expression (Figure 6(a)). We then investigated the role of survivin on the process of platelet-derived mitochondria protecting HUVECs from oxidative stress damage by transferring si-survivin RNA into HUVECs. To confirm the critical role of survivin in mitochondria function, we examined apoptosis levels of interfered HUVECs treated with H_2_O_2_ and SPC by flow cytometry. We found survivin siRNA significantly increased HUVEC apoptosis (Figures 6(b) and 6(c)). Similarly, the caspase-3 activity was significantly increased in the si-survivin+H + SPC (Figures 6(d) and 6(e)). These results confirmed that survivin plays a critical role in platelet-derived mitochondria alleviating apoptosis associated with caspase-3.

### 3.7. Platelet-Derived Mitochondria in SPC Accelerate Wound Healing

**Figure 7:**
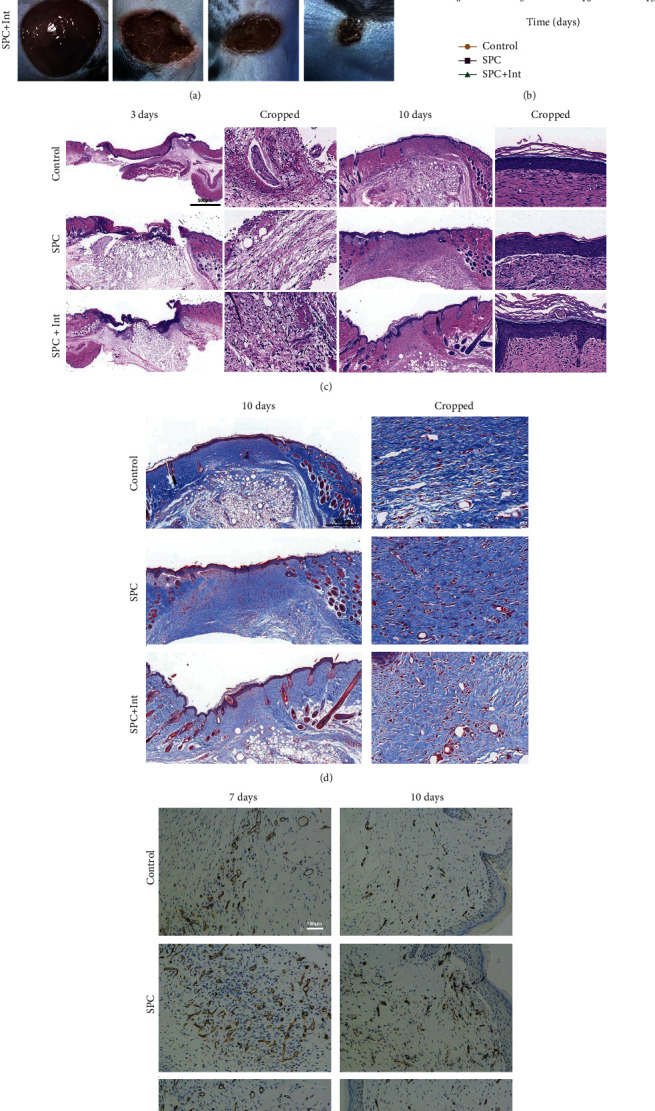
Platelet-derived mitochondria in SPC promoted wound healing. (a) The gross view of wounds closure of mouse treated with PBS, SPC, and SPC + Int at days 0, 3, 7, and 10 and (b) statistical analysis of the wound healing rate (^∗^: control vs. SPC, ^#^: control vs. SPC + Int, and ^&^: SPC vs. SPC + Int). (c) HE staining of regenerated skin tissue in PBS, SPC, and SPC + Int groups at day 3 and day 12. (d) Masson staining of regenerated skin wounds in different groups at day 10. (e) IHC staining of CD31 in wound sections treated with PBS, SPC, and SPC + Int at day 7 and day 10. Significant differences between groups are indicated as ^∗^*p* < 0.05, ^∗∗^*p* < 0.01, ^∗∗∗^*p* < 0.001, ^∗∗∗∗^*p* < 0.0001.

To determine the effects of platelet-derived mitochondria in SPC on skin wound healing, we created full-thickness wound on dorsal skin of mice. The mice were randomly divided into 3 groups treated with PBS, SPC, or SPC + Int. The area of the wounds was measured and photographed on days 0, 3, 7, and 10, and the gross observation of wounds in each group at different time points was displayed (Figures 7(a) and 7(b)). The results showed no signs of infection in the wounds at different degrees. Compared with both the PBS and SPC + Int groups, the wound healing in the SPC group was the best after 3 days, which was generally covered with newborn skin (Figure 7(a)). The wound healing rate was calculated at different times (Figure 7(b)). On days 3 and 7, the SPC group showed higher healing rate compared to the other groups (Figure 7(b)). Especially on day 3, the wound healing rate in the SPC group was the most remarkable better than the other groups (Figure 7(b)). On day 10, there was no statistical difference between the three groups (Figure 7(b)). The results indicated that platelet-derived mitochondria promoted wound closure especially in the early stage. We further evaluated the quality of wound healing by H&E and Masson staining during the repairing process of days 3 and 10 (Figures 7(c) and 7(d)). H&E staining showed that decreased inflammatory infiltration was observed in the SPC on day 3, and more significant thickness of the neoepidermis was observed in the SPC on day 10 (Figure 7(c)). Collagen fibers depositing in the wound determine the quality of tissue remodeling. To study the expression and arrangement of collagen fibers in the wound, we carried out Masson staining. Masson staining showed collagen deposition under the wound was significantly increased in SPC on day 10 (Figure 7(d)), accompanied by an ordered arrangement and uniform density. Finally, we detected the expression of CD31 to explore the effect of platelet-derived mitochondria in SPC on angiogenesis. The results showed that the number of CD31 positive cells was significantly increased in wound tissues of the SPC on day 7 and day 10 (Figure 7(e)).

## 4. Discussion

**Figure 8:**
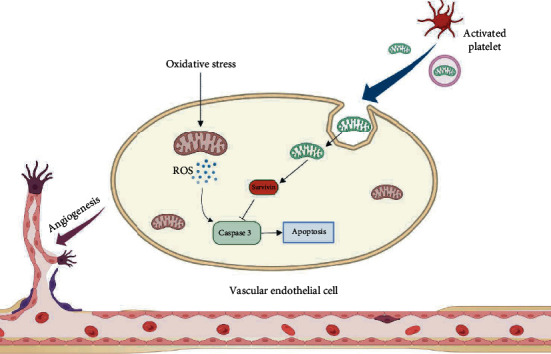
Diagram of our findings that platelet-derived mitochondria in SPC were taken up by HUVECs to reduce the apoptosis caused by oxidative stress. First, platelets were lysed or activated to release exposed or membrane-coated mitochondria. These mitochondria were then absorbed by HUVECs partly by means of dynamin-dependent clathrin-mediated endocytosis. Subsequently, platelet-derived mitochondria attenuated oxidative stress injury of HUVECs by upregulating the expression of survivin. Finally, platelet-derived mitochondria promoted vascularization and wound healing. Created with BioRender.com

In this work, we found that platelets promote wound healing through releasing mitochondria taken up by vascular endothelial cells (Figure 8). Additionally, our experiments provided evidence that platelet-derived mitochondria in SPC can reduce apoptosis of endothelial cells caused by oxidative stress. High-throughput sequencing results hint that the realization of this function is related to the expression of survivin. Further analysis showed that the expression of survivin played an important role in the process of mitochondria derived from platelets alleviating oxidative damage of endothelial cells. Equally important, we excavated the potential of platelets as donor for mitochondrial transplantation and preliminarily determined that ultrasound was used as a suitable method for extracting platelet mitochondria. Overall, our data revealed the importance of platelet mitochondria in protecting endothelial cells from apoptosis and promoting wound healing.

Benefitting from regeneration attribute, platelet concentrates are widely used in clinical practice and trials to promote wound healing and other tissue repair process [28]. However, the mechanism by which platelet lysate promotes wound healing remains unclear. Meanwhile, the method of platelet manipulation is controversial. The early application of platelet concentrates was activated by the mixture of 10% calcium ion solution and bovine thrombin [29, 30]. Due to the most commonly used thrombin which comes from cattle, this raises concerns about safety. In order to find a suitable manipulation method for platelet concentrates, therefore, we chose several manipulation methods including thrombin, freeze-thaw, ultrasound, and photoactivation. We found that ultrasound is the best way to release platelet active materials and mitochondria. This result suggests that ultrasound may be a better way to extract platelet lysate for clinical applications.

Consistent with most clinical applications, we used plasma to suspend platelets [31]. According to the initial cognition, the benefits of platelets are mainly due to the particles contained in platelets, especially *α* particles, which will release a variety of substances after platelets activated [32]. Therefore, several growth factors including PDGF-BB, EGF, FGF-b, and TGF-*β*1 were assayed to characterize these manipulation PCs. Our data revealed the differences of these bioactive molecules in manipulation PCs. FTPC and SPC, prepared from platelet lysate, were enriched growth factors, with significantly higher concentrations in PDGF-BB, EGF, and FGF-b than other manipulation PCs. In particular, SPC were enriched PDGF-BB and FGF-b than FTPC. This may be related to that activating platelets by destroying the cell membrane will release more growth factors. There are studies documented that lysate releases more growth factors, such as BDNF, PDGF-AB, and TGF- *β* [33, 34]. Interestingly, we found that thrombin-promoted PCs produce more TGF-*β*1 than FTPC and SPC. We speculate that platelet degranulation will provide more benefits for TGF-*β*1 release. However, we need more work to explore the mechanism behind this. As we all know, cellular TGF-*β*1 exists in a biologically inactive (latent) form by combing with latency-associated peptide (LAP) [35]. Unexpectedly, our results showed that several manipulation platelet concentrates contain only mature TGF-*β*1. We assume that latent TGF-*β*1 is activated after released from platelets [36, 37]. Numerous other proteins released from platelets, such as GDNF family and EGF family, contain similar structures that affect their biological activity. In addition to the well-known bioactive molecules located in granules, there are a large number of cytoplasmic and membrane proteins that may play a role after platelet activation. However, the remained body of activated platelets limit the release of these proteins. On the contrary, when platelets are lysed, these proteins are more likely to be released to the surrounding environment. To further verify the significance of these differences, we used these manipulation PCs to treat HUVECs. Although SPC had a more significant effect in promoting cell migration, overly complex components in manipulation PCs seem to cause the differences of these molecular unable simply to present in cells. Overall, our results offer strong evidence that SPC is a more noteworthy manipulation platelet concentrates.

Recent work has documented that PCs could alleviate oxidative stress and protect cell from damage [19]. Meanwhile, as a critical member in wound healing, vascular endothelial cells impaired under high levels of ROS would hamper neovascularization [1]. In the context of our study, we wondered that whether SPC reduce the oxidative stress of HUVECs caused by hydroperoxide. Consistent with the above reports, SPC showed exciting benefits including reducing ROS of HUVECs, increasing mitochondrial membrane potential, and reducing cell apoptosis. However, as we mentioned above, the complex components of PCs add to the burden of explaining this phenomenon. Considering the number of released proteins identified varies from 100 to over 800 proteins [38], different platelet concentrates have amazing heterogeneity resulting in different functions or mechanisms. The effect of SPC is likely to partially come from its cytoplasmic proteins, such as superoxide dismutase, glutathione S-transferase, glutathione peroxidase, catalase, thioredoxin reductase, and peroxiredoxin, but thrombin activation platelets may rarely release these proteins. When we use different methods to treat platelet concentrates, we have to rethink the underlying mechanism. Recent work indicated that the presence of growth factors and antioxidative molecule may play a critical role in SPC protecting HUVECs [19]. In addition, several researches provide us with new possibilities that respiration-competent mitochondria can be taken up by recipient cells to save endangered cells [12, 13, 39, 40]. This is an interesting discovery that platelets can release functional mitochondria into membrane-wrapped particles with a size of more than 500 nanometers or as free organelles [16]. Several studies have shown that these mitochondria released by platelets can be “swallowed” by neutrophils, pancreatic *β*-islets, or stem cells where they trigger the unique response path, respectively [16–18]. However, the function of these mitochondria and the intrinsic molecular mechanism are unclear.

In fact, mitochondrial transfer is becoming a promising treatment for clinical application in tissue revitalization [10]. On the one hand, mitochondrial transfer serves as an efficient way to revitalize exhausted cells when they are suffering from intense stress and damage. On the other hand, the intercellular mitochondrial transfer has been reported to contribute to multifunctional cellular activity and have an impact on tumor therapy resistance and inflammation regulation. However, we have to consider the source of mitochondrial donors. Most of the studies performed mitochondria transfer from differentiated cells like fibroblasts, liver cells, MSCs, and others [41]. We consider whether platelets may be a substitute considering that platelets are easier to obtain and easier to purify, and platelets are more likely to release mitochondria after activation. Now, our results showed that platelets are promising donors of mitochondrial transfer in wound healing and provided a feasible method ultrasound to access mitochondrial.

Consistent with the above reports, we found that there were two kinds of mitochondria released from platelets by sonication. In fact, most mitochondria were exposed, not wrapped in vesicles. In addition, it is worth exploring how they enter the cells to function. Generally speaking, there are four routes of mitochondrial transfer from donor cells to recipient cells, including TNT, dendrite, macrovesicles, and internalized without carriers [10]. Interestingly, our study indicated that platelet-derived mitochondria were dominantly internalized by HUVECs via dynamin-dependent endocytosis. This mechanism is similar to that of MSCs internalizing platelet-derived mitochondria [18]. Besides, an analogous mechanism of endocytosis emerged in the transfer of mitochondria from engrafted MSCs to injured lung epithelial cells [42]. Overall, it seems that the transfer of mitochondria is a ubiquitous phenomenon occurring for immunoregulation, tumor, and wound healing, despite underlying molecular machinery and biological effect changing based on the recipient cell types. Interestingly, loss of respiratory function did not prevent mitochondria released from platelets from being absorbed by endothelial cells. On the contrary, endothelial cells treated with hydrogen peroxide swallowed mitochondrial more. One possible explanation is that hydrogen peroxide through the production of metabolites increases vascular endothelial permeability [43].

Given that platelet-derived mitochondria can be engulfed by HUVECs via dynamin-dependent endocytosis, we wondered whether it plays a role in the protective effect of SPC on endothelial cells from oxidative stress. In support of this supposition, exogenous mitochondrial transplantation has been reported to increase cellular viability, decreased ROS, and apoptosis levels [12]. Indeed, our findings established that platelet-derived respiration-competent mitochondria enhanced the viability of endothelial cells.

Interestingly, our documents indicated that platelet-derived mitochondria exerted benefit effect on recipient HUVECs by upregulating gene survivin (also known as *BIRC5*) expression, which is both essential for mitosis and able to inhibit apoptosis [44]. The current perspective is that survivin synergies with XIAP and HBXIP affect the interaction of XIAP with caspases or enlarge the effect of other IAP family members which function on extrinsic apoptotic pathway [45]. Consistent with the above description, the results of high-throughput sequencing showed that platelet-derived mitochondria enhanced cell proliferation and accelerated cell cycle. Previous work documented survivin effect cell cycle by targeting the CPC to the centromeres, dampening microtubule dynamics, and delineating the cleavage plane prior to actomyosin recruitment [44]. At present, the effect of exogenous mitochondria on survivin has not been reported. However, evidence is accumulating that survivin regulates mitochondrial dynamics and metabolism, but exactly how this is achieved also needs to be elucidated [46]. Overall, our results provided new evidence and explanation for the mechanism of exogenous mitochondria acting on recipient cells.

Endothelial cells can be dysfunctional and even die due to the oxidative stress triggered by excessive ROS, thereby delaying the formation of granulation tissue and reepithelialization. Consistent with above results, our animal experiments strongly indicated that platelet-derived mitochondria were benefit for the stimulation of angiogenesis. By regulating the oxidative stress state of endothelial cells, it is a hope to accelerate the cycle of endothelial cells from proliferation to degeneration. The advance of vascularization peak will be beneficial to tissue regeneration and even subsequent tissue remodeling. Similarly, our results showed that platelet-derived mitochondria promoted reepithelialization and increased collagen fiber density. This seems to be that the control of the oxidative stress state of endothelial cells promotes vascularization, thereby improving the speed and quality of wound healing.

While our study showed that platelet-derived mitochondria in SPC are transferred to HUVECs, there was lack of images of the process by which mitochondria are internalized to HUVECs. In addition, we have shown that the protective effect of SPC on HUVECs was blocked by inhibiting the function of mitochondrial in SPC, but we do not know the extra effect of impaired mitochondrial from platelets on HUVECs considering that nonrespiratory mitochondrial was equally internalized by HUVECs. Finally, even though we found the expression of survivin was related to the effect of platelet-derived mitochondria in SPC on HUVECs, further mechanisms need to be explored.

## 5. Conclusion

In conclusion, our results showed that platelet-derived mitochondria were taken up by HUVECs to protect the cells from oxidative stress damage and promote wound healing. What is more, our data provided a new prospective mechanism for exogenous mitochondrial saving dangerous cell via upregulating gene survivin expression. It is equally important that we provided a new donor cell-platelets for clinical mitochondrial transplantation. More importantly, our study provided new strategy to control cell oxidative stress to realize more ideal tissue regeneration or even other disease models, like senescence, though more work is needed.

## Data Availability

The data used to support the findings of this study are available from the corresponding author upon request.

## Abbreviations

ATP: Adenosine triphosphate
ECs: Endothelial cells
EGF: Epidermal growth factor
FGF-b: Fibroblast growth factor
FTPC: Frozen-thawed platelet concentrates
LPC: Light platelet concentrates
MMP: Mitochondrial membrane potential
MIB: Mitochondrial isolation buffer
OCR: Oxygen consumption rate
PCs: Platelet concentrates
PDGF-BB: Platelet-derived growth factor
ROS: Reactive oxygen species
siRNAs: Small interfering RNAs
SPC: Sonicate platelet concentrate
TEM: Transmission electron microscopy
TGF-*β*1: Transforming growth factor
TPC: Thrombin platelet concentrates.

## Supplementary Materials

Supplementary Materials Figure S1: Western blot showed the protein level and the form of TGF-*β*1 in several activated PCs.
