# Supplementary material for: Platelets Facilitate Wound Healing by Mitochondrial Transfer and Reducing Oxidative Stress in Endothelial Cells
Source: Oxid Med Cell Longev. 2023 Feb 20;2023:2345279. doi: 10.1155/2023/2345279 (PMC9970712; doi:10.1155/2023/2345279)
Supplement: Supplementary Materials — Figure S1: Western blot showed the protein level and the form of TGF-β1 in several activated PCs. [file 2345279.f1.docx]

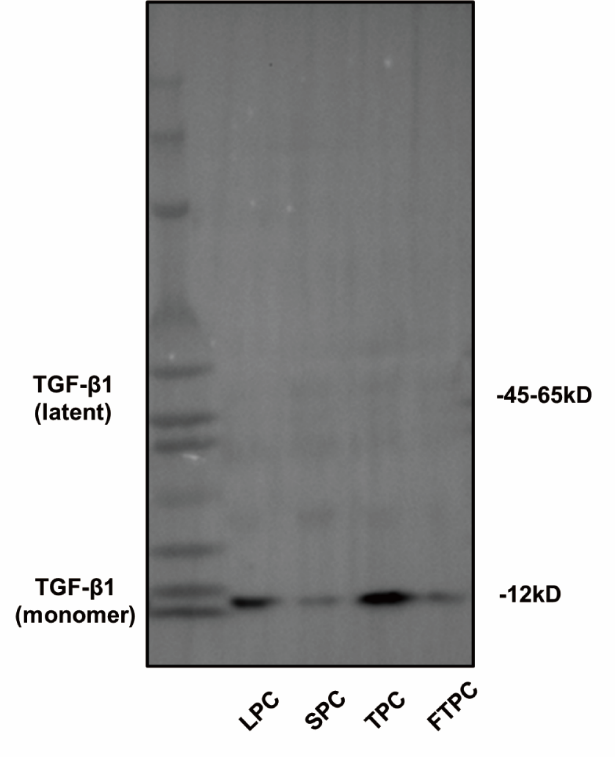


**Figure S1**

**Figure S1: Western blot showed the protein level and the form of TGF-β1 in several activated PCs.**
